# Evaluating active leprosy case identification methods in six districts of Nepal

**DOI:** 10.1186/s40249-023-01153-5

**Published:** 2023-12-06

**Authors:** Ram Kumar Mahato, Uttam Ghimire, Madhav Lamsal, Bijay Bajracharya, Mukesh Poudel, Prashnna Napit, Krishna Lama, Gokarna Dahal, David T. S. Hayman, Ajit Kumar Karna, Basu Dev Pandey, Chuman Lal Das, Krishna Prasad Paudel

**Affiliations:** 1 Epidemiology and Disease Control Division, Department of Health Services, Ministry of Health and Population, Kathmandu, Nepal; 2 Epidemiology and Disease Control Division-Malaria Program Management Unit- SCI-GF, Kathmandu, Nepal; 3 Lalgadh Leprosy Hospital & Service Center, Nepal Leprosy Trust, Lalgadh, Nepal; 4 Molecular Epidemiology and Public Health Laboratory, Infectious Disease Research Centre, Hopkirk Research Institute, Massey University, Palmerston North, New Zealand; 5 Center for Health and Disease Studies-Nepal, Kathmandu, Nepal; 6 DEJIMA Infectious Disease Research Alliance, Nagasaki University, 1-12-4, Sakamoto, Nagasaki, Japan; 7 Leprosy Control & Disability Management Section, EPidemiology and Disease Control Division, DoHS, Kathmandu, Nepal

**Keywords:** Leprosy, Early case detection, Community-based epidemiology, Hidden case, New case detection rate, Attack rate, Cost per case identified, Nepal

## Abstract

**Background:**

Nepal has achieved and sustained the elimination of leprosy as a public health problem since 2009, but 17 districts and 3 provinces with 41% (10,907,128) of Nepal’s population have yet to eliminate the disease. Pediatric cases and grade-2 disabilities (G2D) indicate recent transmission and late diagnosis, respectively, which necessitate active and early case detection. This operational research was performed to identify approaches best suited for early case detection, determine community-based leprosy epidemiology, and identify hidden leprosy cases early and respond with prompt treatment.

**Methods:**

Active case detection was undertaken in two Nepali provinces with the greatest burden of leprosy, Madhesh Province (40% national cases) and Lumbini Province (18%) and at-risk prison populations in Madhesh, Lumbini and Bagmati provinces. Case detection was performed by (1) house-to-house visits among vulnerable populations (*n* = 26,469); (2) contact examination and tracing (*n* = 7608); in Madhesh and Lumbini Provinces and, (3) screening prison populations (*n* = 4428) in Madhesh, Lumbini and Bagmati Provinces of Nepal. Per case direct medical and non-medical costs for each approach were calculated.

**Results:**

New case detection rates were highest for contact tracing (250), followed by house-to-house visits (102) and prison screening (45) per 100,000 population screened. However, the cost per case identified was cheapest for house-to-house visits [Nepalese rupee (NPR) 76,500/case], followed by contact tracing (NPR 90,286/case) and prison screening (NPR 298,300/case). House-to-house and contact tracing case paucibacillary/multibacillary (PB:MB) ratios were 59:41 and 68:32; female/male ratios 63:37 and 57:43; pediatric cases 11% in both approaches; and grade-2 disabilities (G2D) 11% and 5%, respectively. Developing leprosy was not significantly different among household and neighbor contacts [odds ratios (*OR*) = 1.4, 95% confidence interval (*CI*): 0.24–5.85] and for contacts of MB versus PB cases (*OR* = 0.7, 95% *CI* 0.26–2.0). Attack rates were not significantly different among household contacts of MB cases (0.32%, 95% *CI* 0.07–0.94%) and PB cases (0.13%, 95% *CI* 0.03–0.73) (*χ*^2^ = 0.07, *df* = 1, *P* = 0.9) and neighbor contacts of MB cases (0.23%, 0.1–0.46) and PB cases (0.48%, 0.19–0.98) (*χ*^2^ = 0.8, *df* = 1, *P* = 0.7). BCG vaccination with scar presence had a significant protective effect against leprosy (*OR* = 0.42, 0.22–0.81).

**Conclusions:**

The most effective case identification approach here is contact tracing, followed by house-to-house visits in vulnerable populations and screening in prisons, although house-to-house visits are cheaper. The findings suggest that hidden cases, recent transmission, and late diagnosis in the community exist and highlight the importance of early case detection.

**Supplementary Information:**

The online version contains supplementary material available at 10.1186/s40249-023-01153-5.

## Background

Leprosy is a contagious but low pathogenic and chronic infectious disease caused by *Mycobacterium leprae*. It mainly affects peripheral nerves and skin, which results in progressive physical, psychological and social disability in some cases [1, 2]. Disability affects the social and working lives of infected people; social stigma is a significant consequence of leprosy. The first and prime objective of leprosy control programs is to focus on early case detection so that treatment can begin as early as possible after symptoms appear and disability is prevented [3]. In 2022, 174,087 new leprosy cases were reported from 182 countries (21.8 per million population), of which 12 countries reported more than 1000 new cases; the World Health Organization (WHO) South‒East Asia Region (SEAR) accounted for 71.4% of the cases. The new (0.22/10000 population) and child (5.9%) cases, grade 2 disability (G2D, 5.5%) and female (38.9%) case proportions in 2022 indicate ongoing transmission, late diagnosis and underreported cases in females[4, 5].

Nepal has maintained leprosy elimination as a public health problem level at the country level since 2009. However, in 2018, Nepal still reported more than 3200 cases with a registered prevalence of 0.99/10,000 population. Seventeen districts and 3 provinces had a registered leprosy prevalence of > 1/10,000 population, with Madhesh Province (40%) and Lumbini Province (18%) accounting for most cases. The proportions of children, females and G2D cases in Nepal in 2018 were 7.92%, 42% and 4.75%, respectively [6]. The pediatric cases indicate recent transmission, lower female proportions indicate underreporting, and G2Ds suggest late diagnosis, all threatening the elimination status that Nepal achieved in 2009.

The WHO Global Leprosy Strategy 2016–2020 launched in 2016 envisioned accelerated action toward a leprosy-free world. The indicators for this vision were zero children diagnosed with leprosy and visible deformities, a rate of newly diagnosed leprosy patients with visible deformities < 1 per million, and no countries with legislation allowing discrimination on the basis of leprosy. The promotion of voluntary self-reporting is crucial to case detection and for achieving the desired target. The Global Leprosy Strategy 2016–2020, along with the current 2021–2030 strategy, [7] also recommends targeting high-risk and vulnerable groups with increasing active case detection [8]. Active case detection is a more effective strategy that enables early diagnosis and treatment and prevents disability and potentially the spread of infection [9, 10].

Differing approaches are available for different at-risk populations. House-to-house visits of high-risk and vulnerable populations, including people from lower castes such as Dalit, Mushhar and other marginalized communities in Nepal, could identify hidden cases that might transmit the disease in favorable conditions. Contact tracing is a recognized form of active case detection in a group that is significantly more likely to have leprosy than the general population in high- and low-endemic disease burden countries. This form of active detection takes all the registered cases as index cases and trained health workers screen the household and neighboring contacts of those index cases in order to know if the contacts have developed clinical leprosy. Ten percent of the cases diagnosed by trained health workers are re-examined and validated by dermatologists as per National Leprosy Strategy 2021–2025 and Leprosy Post-exposure Program Guidelines [11]. Among different types of contacts, household contacts reportedly have a 3.5 times greater likelihood of having leprosy than social contacts and almost double that of neighbors; however, even social contacts are 2.5 to 3 times more likely to have leprosy than the general population [12]. Studies suggest that the most susceptible populations include family contacts of multibacillary (MB) cases, followed by neighboring contacts and contacts of paucibacillary (PB) cases [13]. However, overcrowding within prisons also makes the prison environment conducive to disease spread. Poor diet, lack of hygiene and physical inactivity are enabling factors; hence, prisoners are more at risk of transmission than the general population [14, 15]. Finally, BCG vaccination, the attenuated bacillus Calmette-Guérin strain of the related *M. bovis* bacteria, reduces leprosy transmission [16, 17].

Here, we use three active case detection methods: (1) house-to-house visits of high-risk and vulnerable populations in Nepali districts with leprosy public health problems; (2) house-to-house visits and examination of contacts of leprosy cases identified between 2 and 5 years ago (retrospective active case finding) [18]; and (3) examination of prisoners to identify early cases in a cross-sectional study. The study also assessed the cost effectiveness of methods to identify active cases and measures of association to highlight key epidemiological features of leprosy in risk areas relevant for control.

## Methods

### Study overview

The general study objective was to determine the epidemiology of leprosy and its protective and risk factors through active and early case detection approaches using three active case detection approaches and to estimate their yield and direct costs. The study was conducted between October and December 2021. The study was performed in three provinces in Nepal: Madhesh Province, Lumbini Province and Bagmati Province. The Siraha and Rauthat districts of Madhesh province and the Banke and Bardia districts of Lumbini Province have been selected as these districts have not eliminated leprosy as a public health problem. These districts have clusters of leprosy cases with ongoing transmission. Only prisons of Bagmati Province were included in study as it was assume that prisoner populations could be at-risk populations. Full details of each study are given below in the "Active case detection" section, Approaches 1–3. Except for the prison population, age and gender were only recorded for cases identified and not other contacts. All cases diagnosed by trained health workers were examined and validated by dermatologists for this study.

### Active case detection

#### Approach 1

House-to-house visits in communities with high-risk groups and vulnerable populations, such as marginalized habitants of Dalit, Mushhar, and Chamar groups, were undertaken in Rautahat District of Madhesh Province and Banke District of Lumbini Province. In Rautahat, four rural municipalities (Palika), namely, Dewahi Gonahi, Rajpur, Ishnath and Rajdevi, were selected in close coordination with district health authorities. These municipalities were considered to have inhabitants from more vulnerable populations. From the four municipalities, 24 sites (wards) covered by 24 health facilities were selected. The same process was followed in Banke, where 27 sites (wards) covered by 27 health facilities from four municipalities, Baijnath, Narainapur, Janaki and Nepalgunj, were selected. A total of 60 to 100 households with inhabitants of marginalized people living in overcrowded houses made of soil or mud, which favored leprosy transmission, were used for the census. Trained local health workers and local female community health volunteers (FCHVs) visited the selected sites and performed house-to-house visits, examining all the members present in the household for any signs of leprosy. In total, 13,420 and 13,049 individuals were examined in Rautahat and Banke, respectively (Additional file 1: Table S1). Local trained health workers examined male individuals, and FCHV examined female individuals present in the household. Simultaneously, demographic and epidemiological variables were collected by a trained local health worker.

Members of the households were informed 2 days before the survey and asked to be present at their own household at the time of the survey via the local FCHV. All suspected cases identified by local health workers and FCHV were invited to health facilities, and cases were confirmed by a dermatologist. After diagnosis confirmation, leprosy cases were treated as per the national protocol.

#### Approach 2

Household and neighboring contacts of previously identified confirmed leprosy cases in the previous 2–5 years were examined in the Siraha district of Madhesh Province and Bardiya of Lumbini Province by trained local health workers. The cases diagnosed between the last 2–5 years in the respective districts were selected randomly in planning meetings conducted before the implementation of field work. Local trained health workers and FCHVs examined 106 and 177 confirmed leprosy case contacts, respectively, in Siraha and Bardiya. A total of 7608 contacts were screened during the case–contact survey (Table 1).

**Table 1:** Leprosy cases and their contacts screened during a case–contact survey

District	Total population [19]	Total index cases	Clinical disease	Index cases	Household contacts^a^	Neighboring contacts^b^	Total screened population
Siraha	739,953	106	MB	53	327	1353	3170
			PB	53	372	118	
Bardiya	459,900	177	MB	107	599	2102	4438
			PB	70	2102	1352	
Total							7608

^a^Household contacts comprised all members > 2 years old residing in the index case household

^b^Neighboring contacts comprised all individuals > 2 years old residing in the nearest 4–6 neighboring houses of the index case

#### Approach 3

Siraha (*n* = 449), Rautahat (*n* = 360), Banke (*n* = 826), Bardiya (*n* = 319), Lalitpur (*n* = 251) and Kathamandu (*n* = 2223) prisons were used as screening sites for active case detection using convenience sampling among 4428 prisoners to assess the transmission status of leprosy in prisons. The prisoner population comprised 4229 males and 199 females.

### Informed consent

In all approaches, participants were requested to give verbal informed consent. As this study was part of regular surveillance of epidemiology and disease control division (EDCD), written informed consent was not obtained. Approval for data publication was obtained from EDCD, and exemption from ethical review (347/2022) was obtained from the ethical board of the Nepal Health Research Council.

### Statistical analysis

Data collected on paper-based questionnaires developed by the Leprosy Control and Disability Management Section (LCDMS)/EDCD were entered in Excel^®^ spreadsheets. Consistency was checked, and data analysis was performed in IBM SPSS statistics 22 (IBM Corp, Armonk, NY, USA) and R version 4.2.0 (R Foundation for Statistical Computing, Vienna, Austria) [20]. Differences in the yields (cases per 100,000 people screened) for all methods (Approaches 1–3) were tested first using prop.test in R and then pairwise using an exact test with a Poisson distribution using the poisson.test in R. The attack rate (AR) with respect to different demographic variables and types of leprosy cases were calculated with 95% confidence intervals (*CI*s) using binomial models in R, where the attack rates are the case per contact calculated from the case-contact survey (Approach 2). Chi-squared tests (*χ*^2^) and odds ratios (*OR*s) using Fisher’s exact test with 95% *CI*s were calculated for associations between attack rates with respect to different demographic variables and between BCG scar presence and leprosy using R’s chisq.test or fisher.test. We used a Poisson generalized linear regression for testing the significance of age classes and gender of being a case from all the case data (Approaches 1–3, see Additional file 1: Table S2), where:

$$\mathrm{log} (E\left({case}_{i}\right))={\beta }_{0}+{\beta }_{Age}{x}_{i}+{\beta }_{Gender}{x}_{j}$$

where ${\beta }_{0}$ is the intercept, ${\beta }_{Age}$ the coefficient for the age class *i* and ${\beta }_{Gender}$ the coefficient for the gender *j*, using R’s glm function. To adjust for the screened population at risk and index cases present in the case-contact survey (Approach 2), we also use simple Poisson regression with an offset for index cases in the population present to assess risk [21], where for district *i*:

$$\mathrm{log} (E\left({new\,cases}_{i}\right))={\beta }_{0}+{\beta }_{district}{x}_{i}+\mathrm{log}\left({index\,cases}_{i}*{population\,screened}_{i}\right)$$

where ${\beta }_{0}$ is the intercept, ${\beta }_{district}$ the coefficient for the district *i*. We also simply offset this with population alone, where the offset was $\mathrm{log}\left({population screened}_{i}\right)$ to test the sensitivity of the results to this assumption.

### Cost analyses

The direct medical and non-medical costs for each approach were calculated and comprised expenses related to training, orientation, health worker per diems, dermatologist’s fees, expenses for monitoring and supervision and data management [22]. The total direct cost was divided by the total number of patients identified or diagnosed by the approach and was derived per unit cost for the leprosy cases identified. Finally, for discussion, we converted costs from national currencies to US dollars for comparison. We used the date in publications and adjusted to 25 December 2021 rates using Google’s default currency convertor provided by Morningstar at 119.11 Nepalese rupee (NPR) per US dollar (USD).

## Results

### Comparison of different approaches of active case detection

New leprosy cases were identified during house-to-house visits (*n* = 27), contact tracing (*n* = 19) and prison screening (*n* = 2) from a total of 38,505 screened people (Table 2). New case detection rates were highest in contact tracing (250 per 100,000 population), followed by house-to-house visits (102 per 100,000) and prison screening (45 per 100,000). We found statistically significant differences in the yields (*χ*^2^ = 169, *df* = 2, *P* < 0.001), with significant differences between all approaches, where contact tracing was greater than both house-to-house visits (rate ratio 2.45, 95% *CI* 1.94–3.12, *P* < 0.001) and prison screening (rate ratio = 5.56, 95% *CI* 4.03–7.81, *P* < 0.001) and house-to-house visits greater than prison screening (rate ratio = 2.27, 95% *CI* 1.58–3.29, *P* < 0.001). However, house-to-house visits were the cheapest cost per case identified at NPR 76,500/case (USD 642), followed by contact tracing (NPR 90,286/case; USD 758) and prison screening (NPR 298,300/case; USD 2504).

**Table 2:** Comparison of different approaches of active case detection giving the approach, numbers screened, numbers of confirmed cases, and the clinical classification, gender, pediatric numbers, grade and costs of those cases

Approach	Screened	Suspected cases	Confirmed cases	New case detection rate (/100,000)	PB:MB (%)	F:M (%)	Pediatric cases (%)^a^	New leprosy cases with grade 2 disabilities (%)	Cost/case NPR (USD)
House-to-house visits	26,469	365	27	102	16:11 (59:41)	17:10 (63:37)	3 (11)	3 (11)	76,500 (USD 642)
Contact tracing	7608	214	19	250	13:6 (68:32)	11:8 (57:43)	2 (11)	1 (5)	90,286 (USD 758)
Prison screening	4428	185	2	45	0:2 (0:100)	0:2 (0:100)	0 (0)	0 (0)	298,300 (USD 2504)

Screened: Total numbers screened; PB/MB: paucibacillary/multibacillary ratio; F/M: Female/Male ratio; G2D: Grade 2 deformity.

^a^A breakdown of these at the district level given in Additional file 1: Table S3

Just two MB cases were discovered in adult male prisoners. House-to-house and contact tracing case PB:MB ratios were 59:41 and 68:32; Female/Male ratios 63:37 and 57:43; pediatric cases 11% in both approaches; and G2D 11% and 5%, respectively.

### Age and gender of leprosy cases

**Fig. 1:**
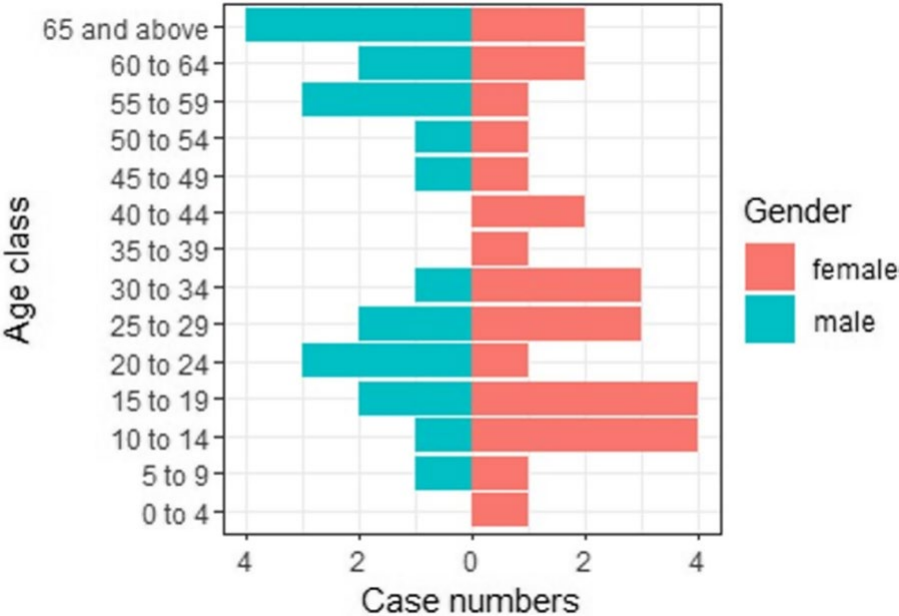
Age and gender of identified leprosy cases

In aggregate, the number of females among confirmed leprosy cases (28/48) was higher than that of males, but the difference was not significant (58%, 95% *CI* 0.43–0.72, *P* = 0.58) (Fig. 1, Additional file 1: Table S2). Neither age (*P* = 0.55) nor gender (*P* = 0.57) was significant using a Poisson regression model.

### Attack rate and associations of contacts and leprosy

Individuals with a history of two to five years of proximate contact with confirmed leprosy cases were examined, in which attack rates were higher among household contacts of MB cases (0.32%, 95% *CI* 0.07–0.94) than among neighboring contacts of the same cases (0.23%, 0.1–0.46), but this difference was not significant (*χ*^2^ = 0.07, *df* = 1, *P* = 0.9). Neighboring contacts of the PB cases were found to have a higher attack rate (0.48%, 95% *CI* 0.19–0.98) compared to the household contact (0.13%, 95% *CI* 0.03–0.73) of the same cases, but this was again not significant (*χ*^2^ = 0.8, *df* = 1, *P* = 0.7) (Additional file 1: Fig. S1, Table S4).

Differences in related associations, such as household contacts of MB cases being a case compared to neighbors (*OR* = 1.4, 95% *CI* 0.24–5.85, *P* = 0.71) or PB household cases (*OR* = 2.5, 95% *CI* 0.2–129.1, *P* = 0.63) and other variations of these associations were not significantly different. Further data and tables are provided in the supplementary information (Additional file 1: Fig. S2, Table S5).

The contact tracing of 106 and 177 leprosy index cases in Siraha (*n* = 3170) and Bardiya (*n* = 4438) after 2–5 years of proximate contact with index cases identified 14 new cases in Siraha (0.13 cases per index case, 95% *CI* 0.07–0.21) and 5 in Bardiya (0.03, 95% *CI* 0.01–0.06). This was statistically significant (*χ*^2^ = 9.8, *df* = 1, *P* = 0.002), including adjusting for index cases and screened contacts (*β* = 1.9, standard error = 0.5, *P* < 0.001). The result was insensitive to offsetting the screening population (*β* = 1.4, standard error = 0.5, *P* < 0.01).

### Gender, case classification and disability

We found no significant differences between gender and advanced (MB) leprosy (*OR* = 0.47, 95% *CI* 0.11–1.78, *P* = 0.24) or G2D (*OR* = 0.76, 95% *CI* 0.05–11.4, *P* = 1) using all 48 newly confirmed cases.

### BCG and leprosy transmission

Among the total participants, 569 visited a health facility for confirmation of leprosy and were also inspected for a BCG vaccination scar by a dermatologist. Those participants with the presence of a BCG scar were found to have significantly lower odds of having leprosy (18 of 341, Table S5, Table S6) than those without (27 of 201), with an *OR* = 0.42 (95% *CI* 0.22–0.81, *P* = 0.007).

## Discussion

We report 48 new leprosy cases from 38,505 screened people, comprising 29 from house-to-house screening among vulnerable populations, 19 from case-contact tracing and 2 from prisoner screening. House-to-house screening and contact tracing discovered 11% of pediatric cases in both approaches and 11% and 5% of G2D cases, respectively, indicating new transmission events and late diagnosis, highlighting the gaps in leprosy control programs.

The new case detection rate was highest in contact tracing (250), followed by house-to-house visits (102) and prison screening (45) per 100,000 population screened, with all differences significant (*P* < 0.001; see “Results” section), whereas the most cost-efficient approach here was house-to-house visits (NPR 76,500/case; USD 642/case), followed by contact tracing (NPR 90,286/case; USD 758/case) and prison screening (NPR 298,300/case; USD 2540/case). These costs per case detected are similar to those reported for other countries; for example, case contact tracing was approximately USD 529 (vs ~ USD 758) in a similar study of Nigeria [23], scaled by inflation and using December 2021 exchange rates. The effectiveness and cost efficiency suggest that implementing both approaches in parallel may be optimal.

The epidemiological and clinical features of the identified confirmed cases were not significantly different. This is possibly because of the small sample of confirmed cases. The PB:MB ratio differed from the global status (35:65), but greater sample sizes might alter this. Similarly, the female:male ratio differed, but with large sample sizes, it might change to match the national (42:58) and global (40:60) ratios. However, if the findings here are true but simply lack statistical power due to smaller sample sizes, then these altered ratios could be due to active case detection versus passive case detection and suggest that females with leprosy are often hidden with passive surveillance [4]. However, details on gender and age were only recorded for cases, so differences in gender and age rates are not available but could be useful for future efforts. Studies have highlighted that leprosy cases are underreported [24], along with methods to address underreporting statistically [25, 25–27], but future efforts should also aim for earlier detection through active case detection and to reduce the stigma attached to leprosy so that cases are not hidden.

The transmission attack rates observed in household (0.32%, 95% *CI* 0.07–0.94) and neighboring (0.13%, 95% *CI* 0.03–0.73) contacts of MB cases and household (0.23%, 95% *CI* 0.1–0.46) and neighboring (0.48%, 95% *CI* 0.19–0.98) contacts of PB cases were not significantly different. The rates, however, are lower than some other reports, such as 2% in Brazil in 2008 [17]. This requires additional studies in more districts to confirm, but the lower attack rate in the current Nepalese situation also indicates progress toward elimination of the disease. The lack of a significant difference in household contacts of cases developing leprosy compared to neighboring contacts (0.78, 95% *CI* 0.19–2.45) differs from other findings where household contacts may have twice the risk of developing disease compared to neighboring contacts [12, 28]. The reasons for this could be sample size and statistical power or that other factors are either reducing the within-household transmission or increasing the neighbor-case transmission. Again, further work is needed to determine which is occurring, but if within-household transmission is reduced, this could be a sign of successful case management. The use of genomic epidemiology may help elucidate transmission chains [29].

Contact tracing of leprosy index cases was conducted after 2–5 years of proximate contact in both Siraha (Madhesh Province) and Bardiya (Lumbini Province), with more new cases per index case in Siraha (14, 0.13 cases per index case, 95% *CI* 0.07–0.21) than in Bardia (5, 0.013 per case, 95% *CI* 0.01–0.06). The difference was significant, including adjusting for index cases and screened populations. It was reported that leprosy postexposure prophylaxis (LPEP) has been implemented in Bardia for several years.

A further encouraging finding was that BCG vaccination with the presence of a scar had a significant protective effect against leprosy (*OR* = 0.42, 95% *CI* 0.22–0.81). This finding is consistent with other findings, such as in Brazil, where the *OR* = 0.27 (95% *CI* 0.13–0.59) [17]. The findings suggest that BCG immunization programs will successfully contribute to leprosy elimination. For Nepal, this is encouraging because BCG is given at birth and national coverage is high at 97.8% (95% *CI* 95.8–98.7) for BCG [30, 31]. However, like all immunization programs, there are often small pockets of people where there is lower vaccine coverage, and lower BCG is reported for at-risk populations such as Madhesi, Dalit, and some religious minorities, who were targeted for screening here [32]. Future immunization programs should aim to ensure that at-risk communities are reached to achieve leprosy elimination goals.

Finally, our findings of the study revealed that hidden cases in the community can be identified and treated by active case detection approaches, and these approaches are now included in the Nepali National Leprosy Strategy 2021–2025.

Our study has several limitations, but the key ones include that the study was carried out in the risk population for leprosy transmission, so the findings are limited to these and not applicable to the general population. The costs applied per case identified were calculated in geographically privileged population, and hence will differ from those in more geographically difficult terrain, like mountainous regions. It is also possible that some members of households, and so cases, were missed, despite best efforts, impacting calculations.

## Conclusions

The new case detection rates identified in this study suggest sustained levels of transmission in the communities screened. The proportion of pediatric cases (> 10%) is evidence of recent transmission, and the proportion of G2D confers evidence of late diagnosis and inadequate surveillance in the community. Although not significant, the Female:Male case ratio being the reverse of the global and national reports from passive case surveillance systems indicates hidden cases in the community, suggesting that active surveillance is required to hasten leprosy elimination. The reduced attack rate compared to earlier studies, however, suggests some progress toward disease elimination, and BCG vaccination should be given more attention as a tool for elimination, as it reduces transmission. Together, active case detection through house-to-house visits and contact tracing to detect early and hidden cases, along with the optimal use of BCG, might help reduce transmission, prevent disabilities, and move Nepal closer toward elimination.

### Supplementary Information

**Additional file 1:** **Table S1.** Number of households and individuals screened for active case detection. **Table S2.** Age and sex description of leprosy cases identified during active case detection. **Table S3.** Attack rate in contacts of leprosy. See also Fig. S1. **Table S4.** Odds ratios for associations using Fisher’s exact test. See also Fig. S2. **Table S5.** Impact of BCG on leprosy transmission. **Figure S1.** Attack rates from case-contact surveys. Ninety-five percent confidence intervals (95% *CI*) are shown. **Figure S2.** Odds ratios (*OR*) for associations among cases and contacts. Estimates are shown with 95% confidence intervals (*CI*). Intervals overlapping *OR* = *1* are not significantly different. Note the log10 y-axis because of wide confidence intervals.

## Data Availability

The datasets generated and/or analyzed during the current study are not publicly available because the individual privacy of patients could be compromised but are available from the corresponding author on reasonable request.

## Abbreviations

BCG: Bacille Calmette-Guerin
*CI*: Confidence interval
EDCD: Epidemiology and Disease Control Division
FCHV: Female community volunteer
G2D: Grade-2 disabilities
LCDMS: Leprosy Control and Disability Management
LPEP: Leprosy postexposure prophylaxis
MB: Multibacillary
NPR: Nepalese rupee
*OR*: Odds ratios
PB: Paucibacillary
SEAR: South‒East Asia Region
SPSS: Statistical Package for the Social Sciences
WHO: World Health Organization

## References

[1] Bhat RM, Prakash C (2012). Leprosy: an overview of pathophysiology. Interdiscip Perspect Infect Dis..

[2] Joseph GA, Sundar Rao PSS (1999). Impact of leprosy on the quality of life. Int J Lepr Other Mycobact Dis.

[3] World Health Organization (2020). Leprosy/Hansen disease: management of reactions and prevention of disabilities.

[4] Harrell GT (1947). Epidemiology of leprosy. J Am Med Assoc.

[5] WHO. Weekly epidemiological record. Vol. 98. 2023.

[6] Department of Health services (DoHS). Annual Report: Department of Health Services 2075/76 (2018/19). Vol. 76, Department of Health Services, Ministry of Health and Population, Government of Nepal. 2019. https://publichealthupdate.com/department-of-health-services-dohs-annual-report-2075-76-2018-19/. Accessed 27 Feb 2021.

[7] WHO (2021). Towards zero leprosy Global Leprosy (Hansen’s disease) strategy 2021–2030.

[8] WHO (2016). Global leprosy strategy 2016–2020: accelerating towards a leprosy-free world. Wkly Epidemiol Rec..

[9] Moura MLN, Dupnik KM, Sampaio GAA, Nóbrega PFC, Jeronimo AK, do Nascimento-Filho JM (2013). Active Surveillance of Hansen’s disease (Leprosy): importance for case finding among extra-domiciliary contacts. PLoS Negl Trop Dis..

[10] Tiendrebéogo A, Sow SO, Traore M, Sissoko K, Coulibaly B (1999). Comparison of two methods of leprosy case finding in the circle of Kita in Mali. Int J Lepr Other Mycobact Dis.

[11] Government of Nepal (2023). Epidemiology and diseases control division (EDCD). National Leprosy Strategy 2021–2025.

[12] WHO (2020). Leprosy/Hansen disease: contact tracing and post-exposure prophylaxis.

[13] de Campos DCC, Dutra APB, Suares VL, de Carvalho PAC, Camargo LMA (2015). New strategies for active finding of leprosy cases in the Amazonian region. Rev Soc Bras Med Trop.

[14] Vijayakumaran P, Mahipathy PV, Misra RK, Petro TS, Ramanujan R, Karunakaran S (1997). Hidden cases of leprosy (in prison). Indian J Lepr..

[15] Bernardes Filho F, Santana JM, de Almeida RCP, Voltan G, de Paula NA, Leite MN (2020). Leprosy in a prison population: a new active search strategy and a prospective clinical analysis. PLoS Negl Trop Dis.

[16] Merle CS, Cunha SS, Rodrigues LC (2010). BCG vaccination and leprosy protection: review of current evidence and status of BCG in leprosy control. Expert Rev Vaccines.

[17] Goulart IMB, Bernardes Souza DO, Marques CR, Pimenta VL, Gonçalves MA, Goulart LR (2008). Risk and protective factors for leprosy development determined by epidemiological surveillance of household contacts. Clin Vaccine Immunol.

[18] Fürst T, Cavaliero A, Lay S, Dayer C, Chan S, Smrekar A (2017). Retrospective active case finding in Cambodia: an innovative approach to leprosy control in a low-endemic country. Acta Trop.

[19] NSO. National statistics office. National population and housing census 2021, vol 39. 2021. https://nepalindata.com/resource/NATIONAL-REPORT--NATIONAL-POPULATION-AND-HOUSING-CENSUS-2021/. Accessed 19 Oct 2023.

[20] R Core Team. A language and environment for statistical computing. R foundation for statistical computing. 2023. https://www.R-project.org. Accessed 16 Sep 2023.

[21] Tim CE (1986). Epidemiologic programs for computers and calculators: decision-tree analysis using a microcomputer. Am J Epidemiol.

[22] Hayman DTS, Marshall JC, French NP, Carpenter TE, Roberts MG, Kiedrzynski T (2017). Cost-benefit analyses of supplementary measles immunisation in the highly immunized population of New Zealand. Vaccine.

[23] Ezenduka C, Post E, John S, Suraj A, Namadi A, Onwujekwe O (2012). Cost-effectiveness analysis of three leprosy case detection methods in Northern Nigeria. PLoS Negl Trop Dis..

[24] De Oliveira GL, Oliveira JF, Pescarini JM, Andrade RFS, Nery JS, Ichihara MY (2021). Estimating underreporting of leprosy in brazil using a bayesian approach. PLoS Negl Trop Dis.

[25] de Oliveira GL, Loschi RH, Assunção RM (2017). A random-censoring Poisson model for underreported data. Stat Med.

[26] Chen J, Song JJ, Stamey JD (2022). A Bayesian hierarchical spatial model to correct for misreporting in count data: application to state-level COVID-19 data in the United States. Int J Environ Res Public Health..

[27] de Oliveira GL, Argiento R, Loschi RH, Assunç˜ao RM, Ruggeri F, Branco MD (2022). Bias correction in clustered underreported data. Bayesian Anal..

[28] WHO. Guidelines for the diagnosis, treatment and prevention of leprosy. OMS. 2018;1–87.

[29] Cancino-Muñoz I, López MG, Torres-Puente M, Villamayor LM, Borrás R, Borrás-Máñez M (2022). Population-based sequencing of Mycobacterium tuberculosis reveals how current population dynamics are shaped by past epidemics. Elife.

[30] Government of Nepal M of H and P. National Immunization Programme. Available from: https://www.mohp.gov.np/eng/program/child-health-services/nip. Accessed 18 Jul 2022.

[31] Rauniyar SK, Iwaki Y, Yoneoka D, Hashizume M, Nomura S (2021). Age-appropriate vaccination coverage and its determinants in children aged 12–36 months in Nepal: a national and subnational assessment. BMC Public Health.

[32] Shrestha S, Shrestha M, Wagle RR, Bhandari G (2016). Predictors of incompletion of immunization among children residing in the slums of Kathmandu valley, Nepal: a case-control study. BMC Public Health.

