# Supplementary material for: Evaluating active leprosy case identification methods in six districts of Nepal
Source: Infect Dis Poverty. 2023 Dec 6;12:111. doi: 10.1186/s40249-023-01153-5 (PMC10698884; doi:10.1186/s40249-023-01153-5)
Supplement: Supplementary file 1 — Additional file 1: Table S1. Number of households and individuals screened for active case detection. Table S2. Age and sex description of leprosy cases identified during active case detection. Table S3. Attack rate in contacts of leprosy. See also Fig. S1. Table S4. Odds ratios for associations using Fisher’s exact test. See also Fig. S2. Table S5. Impact of BCG on leprosy transmission. Figure S1. Attack rates from case-contact surveys. Ninety-five percent confidence intervals (95% CI) are shown. Figure S2. Odds ratios (OR) for associations among cases and contacts. Estimates are shown with 95% confidence intervals (CI). Intervals overlapping OR = 1 are not significantly different. Note the log10 y-axis because of wide confidence intervals. [file 40249_2023_1153_MOESM1_ESM.docx]

**Tables**

Table S1. Number of households and individuals screened for active case detection

| District | Total population | Palika (municipality) | Households screened | People screened |
| --- | --- | --- | --- | --- |
| Rautahat | 813,573 | Dewahi-Gonahi | 400 | 2744 |
|  |  | Rajpur | 497 | 3238 |
|  |  | Ishnath | 300 | 1958 |
|  |  | Rajdevi | 787 | 5480 |
| Banke | 603,194 | Nepalgunj | 891 | 5532 |
|  |  | Narainapur | 418 | 2229 |
|  |  | Janaki | 585 | 3612 |
|  |  | Baijnath | 355 | 1676 |
| Total |  |  | 4233 | 26,469 |

Table S2. Age and sex description of leprosy cases identified during active case detection

| Years | Total cases | Number of female |
| --- | --- | --- |
| 0 to 4 | 1 | 1 |
| 5 to 9 | 2 | 1 |
| 10 to 14 | 5 | 4 |
| 15 to19 | 6 | 4 |
| 20 to 24 | 4 | 1 |
| 25 to 29 | 5 | 3 |
| 30 to 34 | 4 | 3 |
| 35 to 39 | 1 | 1 |
| 40 to 44 | 2 | 2 |
| 45 to 49 | 2 | 1 |
| 50 to 54 | 2 | 1 |
| 55 to 59 | 4 | 1 |
| 60 to 64 | 4 | 2 |
| 60 and above | 6 | 2 |
| Overall | 48 | 27 |

Table S3: Attack rate in contacts of leprosy. See also Figure S1.

| Type of Contact | Cases/number of contacts | Attack rate (%, 95% confidence intervals) |
| --- | --- | --- |
| Household contact of MB cases | 3/926 | - 1. (0.07 - 0.94) |
| Household contact of PB cases | 1/757 | 0.13 (0.03 - 0.73) |
| Neighbouring contact of MB cases | 8/3455 | 0.23 (0.1 - 0.46) |
| Neighbouring contact of PB cases | 7/1470 | 0.48 (0.19 - 0.98) |

Table S4. Odds ratios for associations using Fisher's exact test. See also Figure S2.

| Association | Odds ratio (95% *CI*) | *P*-value |
| --- | --- | --- |
| MB cases: Household vs neighbour | 1.4 (0.24 - 5.85) | 0.71 |
| PB cases: Household vs neighbour | 0.28 (0.01 - 2.17) | 0.28 |
| All cases: Household vs neighbour | 0.78 (0.19 - 2.45) | 0.8 |
| Household cases: MB vs PB | 2.5 (0.2 - 129.1) | 0.63 |
| Neighbour cases: MB vs PB | 0.49 (0.15 - 1.57) | 0.16 |
| All cases: MB vs PB | 0.7 (0.26 - 2.0) | 0.47 |

Table S5. Impact of BCG on leprosy transmission

| BCG scar | Leprosy | No Leprosy |
| --- | --- | --- |
| Present | 18 | 323 |
| Absent | 27 | 201 |

**Figures**

Figure S1. Attack rates from case-contact surveys. Ninety-five percent confidence intervals (95% *CI*) are shown.


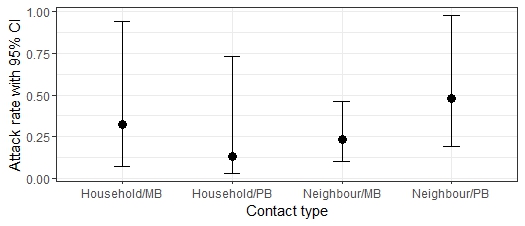


MB – multibacillary; PB – paucibacillary; Household – household contact; Neighbour – neighbouring house contact.

Figure S2. Odds ratios (*OR*) for associations among cases and contacts. Estimates are shown with 95% confidence intervals (*CI*). Intervals overlapping *OR=1* are not significantly different. Note the log10 y-axis because of wide confidence intervals.
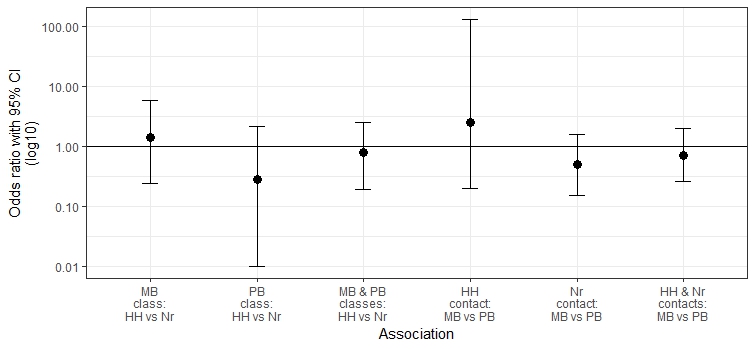


MB – multibacillary; PB – paucibacillary; Household – household contact; Neighbour – neighbouring house contact.
